# Phase fMRI Reveals More Sparseness and Balance of Rest Brain Functional Connectivity Than Magnitude fMRI

**DOI:** 10.3389/fnins.2019.00204

**Published:** 2019-03-18

**Authors:** Zikuan Chen, Zening Fu, Vince Calhoun

**Affiliations:** ^1^The Mind Research Network and LBERI, Albuquerque, NM, United States; ^2^Department of Electrical and Computer Engineering, University of New Mexico, Albuquerque, NM, United States

**Keywords:** magnitude and phase fMRI, independent component analysis (ICA), functional network connectivity (FNC), functional connectivity sparseness, functional connectivity balance

## Abstract

Conventionally, brain function is inferred from the magnitude data of the complex-valued fMRI output. Since the fMRI phase image (unwrapped) provides a representation of brain internal magnetic fieldmap (by a constant scale difference), it can also be used to study brain function while providing a more direct representation of the brain's magnetic state. In this study, we collected a cohort of resting-state fMRI magnitude and phase data pairs from 600 subjects (age from 10 to 76, 346 males), decomposed the phase data by group independent component analysis (pICA), calculated the functional network connectivity (pFNC). In comparison with the magnitude-based brain function analysis (mICA and mFNC), we find that the pFNC matrix contains fewer significant functional connections (with *p*-value thresholding) than the mFNC matrix, which are sparsely distributed across the whole brain with near/far interconnections and positive/negative correlations in rough balance. We also find a few of brain rest sub-networks within the phase data, primarily in subcortical, cerebellar, and visual regions. Overall, our findings offer new insights into brain function connectivity in the context of a focus on the brain's internal magnetic state.

## Introduction

Phase functional magnetic resonance imaging (fMRI) is an MRI technique dedicated to fMRI phase data acquisition and post-acquisition processing and analysis. In principle, an fMRI study produces a timeseries of complex-valued images consisting of pairwise magnitude and phase components; therefore, the fMRI phase data are generated together with the magnitude data in an fMRI experiment (at no extra cost). Since the complex-valued fMRI data (magnitude and phase images in pairs) are formed from the same magnetic source (the internal inhomogeneous magnetic fieldmap) through intravoxel dephasing signal detection and subsequent complex modulo/argument calculations (Chen and Calhoun, 2015b), both are useful for brain function depiction with different representations (in different measurements). In theory, the fMRI phase data are more suitable for brain function analysis since phase imaging represents the brain magnetic state seen in internal magnetic fieldmaps.

There is a body of reports on the exploration and exploitation of phase fMRI for brain function study (Rowe, 2005, 2009; Arja et al., 2009; Feng et al., 2009; Balla et al., 2014; Bianciardi et al., 2014; Chen and Calhoun, 2016b; Ozbay et al., 2016). Under linear imaging conditions, an fMRI phase image represents the brain internal magnetic field distribution captured at a timepoint (Chen and Calhoun, 2015b; Chen et al., 2018b). This portrays a brain magnetic state (a magnetization state in a main field B_0_, in preparation for MRI scanning) during brain activity (Shmueli et al., 2009; Li et al., 2011; Chen and Calhoun, 2013, 2015a; Wang and Liu, 2015).

In theory, the fMRI magnitude signal is calculated from the complex signal by a nonnegative nonlinearity (e.g., |±1| = 1) that fails to represent the source of an internal fieldmap (e.g., degenerating the signs associated with the bipolar-valued field distribution). In comparison, the fMRI phase signal is calculated from the complex MRI signal through a trigonometric operation, *arctan*(φ), which can be linearly approximated by *arctan*(φ) ≈ φ for |φ| < 1 (φ denotes a phase signal, measured in units of radian, related to the field value by a constant scale γT_E_). Therefore, a phase image is linearly related to the magnetic field in linear phase fMRI (Haacke et al., 1999; Chavhan et al., 2009; Chen and Calhoun, 2015b, 2016b). We may infer the internal fieldmap source from an fMRI phase image under linear approximation; however, such inverse mapping is not available from fMRI magnitude data (due to an irreversible magnitude nonlinearity like |±1| = 1).

The trigonometric *arctan*(φ) gives a good linearization for a very small φ, as mathematically defined by *arctan*(φ) = φ for |φ*|* < < 1. In order to maximally reduce the nonlinearity associated with *arctan*(φ), we adopt an additive perturbation model, φ(t) = φ_0_ ± δφ(t), to extract the BOLD-only phase signal (the perturbation term δφ(t)) from a timeseries of BOLD phase signals through complex division (a Hilbert inner product) (Chen and Calhoun, 2016b; Chen et al., 2018b). The BOLD-only phase signal δφ results in good linear mapping of the source of BOLD-only magnetic field perturbation by reducing the nonlinearity associated with *arctan*(φ).

Given a timeseries of fMRI images, we can break down brain function into a collection of brain subfunctions (subnetworks) through an independent component analysis (ICA) method (Calhoun et al., 2001; McKeown et al., 2003; Guo and Pagnoni, 2008; Calhoun and Adali, 2012). Taking advantage of data-driven multivariate statistics, the ICA method has been successfully extended to allow population-level group data analysis (a technique of group ICA) (Calhoun et al., 2001; Beckmann et al., 2005; Guo and Pagnoni, 2008; Calhoun and Adali, 2012). Accordingly, we can apply group ICA to magnitude and phase data separately for brain function decompositions, as denoted by mICA and pICA (Chen et al., 2018a). For comparison of mICA and pICA in correspondence, we constrain the pICA with the magnitude-inferred group information and implement group-information-guided (GIG) pICA (Du and Fan, 2013).

Using mICA and pICA, we then calculate their functional network connectivity (FNC) matrices (denoted by mFNC and pFNC) based on the temporal correlation of mICA and pICA timecourses (Jafri et al., 2008; Arbabshirani and Calhoun, 2011). For larger population data analysis, we may discard the insignificant functional connections based on statistical significance (based on *p*-value assessment). For example, a *p*-value thresholding (<10^−10^) removes insignificant connections in mFNC, enhancing identification of significant whole-brain connections such as sparsity, balance, and near and far couplings.

We have recently reported on a method of comparing magnitude and phase-based brain functional connectivity in the resting state via statistical analysis over 100 subjects (Chen et al., 2018a). This revealed interesting similarities and distinctions between mFNC and pFNC. Here, we used a larger cohort of subject data (*N* = 600) to analyze the brain functional connectivity patterns in mFNC and pFNC matrices. We addressed the following aspects: intra-domain (short-range, near) and inter-domain (long-range, far) connections, positive and negative connections, sparseness and nonuniformity of connection distribution, and robustness and significance of group-level connections.

## Methods

### Data Collection

A collection of 600 subject datasets (in pairs of magnitude and phase images) were acquired from a cohort of participants (age: 10–76 years, 346 male/254 female) by subject scanning in a Siemens TrioTim 3T scanner at the Mind Research Network. Informed consent was obtained for each subject and the subject scanning protocol was approved by the IRB at the University of New Mexico. The data were gained from the subjects anonymously prior to group analysis.

The fMRI experiments were performed with the following parameter settings: 12-channel coil, GRE-EPI sequence, T_E_ = 29 ms, T_R_ = 2 s, flip angle = 75°, field of view = 240 cm × 240 cm, matrix size = 64 × 64, voxel size = 3.75 mm × 3.75 mm × 4.55 mm, slice thickness = 3.5 mm, slice gap = 1.05 mm, total slices 33, acquisition time (T_A_) = 5 min, and total volumes 150. Subjects were instructed to keep their eyes open during the scanning and fixate on a foveally presented cross. We obtained two groups of fMRI data, using magnitude and phase images in pairs, with each in a 4D format (64 × 64 × 33 × 150, 3D spatial and 1D temporal in dimension).

### Data Processing

Preprocessing the fMRI magnitude images included removing the first two timepoints to avoid T1 equilibration effects; realignment using INRIalign; slice-timing correction using the middle slice as the reference frame; spatial normalization into MNI space with resampled isotropic voxels (3 × 3 × 3 mm); and spatial smoothing with a Gaussian kernel (FWHM = 9 mm). Through data processing, each 4D subject data (magnitude and phase separately) in 64 × 64 × 33 × 150 format was converted to 53 × 63 × 46 × 148. For fMRI magnitude image preprocessing, we used the SPM8 automated pipeline (Chen et al., 2018a) as reported (http://www.fil.ion.ucl.ac.uk/spm/software/spm8/).

### Extracting BOLD-Only Phase Signals

The raw phase images were first converted to a range in radian (–π, π) and denoted by φ (bipolarly valued). Then, the phase series images were subjected to spatial realignment through the 3D affine transformation using the motion correction parameters (4 × 4 affine transformation) as derived from the magnitude image realignment in the corresponding magnitude timeseries. Upon phase timeseries image realignment, a complex division (Equation A2 in Appendix) was used to extract the temporal phase changes (BOLD-only phase response) with respect to the middle frame at the middle timepoint in the series (Chen et al., 2018b), as denoted by δφ (bipolarly valued). This calculation is a time-domain phase-unwrapping technique that can extract the small temporal phase changes (< < π) buried in phase-wrapped timeseries signals (Chen and Calhoun, 2016b). Using the phase image processing, we obtained a 4D phase data δφ(**r**,t) for each subject in a format of 53 × 63 × 46 × 148.

### Group mICA and GIG-pICA

The SPM-processed magnitude data were decomposed into functional networks using a group-level spatial ICA as illustrated (Chen et al., 2018a) and implemented in the GIFT toolbox (http://mialab.mrn.org/software/gift/). We decomposed the group magnitude data into a number of 100 brain subfunctions (a relatively high model order brain functional ICA), denoted by mICA. The Infomax spatial ICA algorithm was repeated 10 times in ICASSO (http://www.cis.hut.fi/projects/ica/icasso). The aggregate spatial maps were estimated as modes of spatiotemporal ICA(**r**, t). Subject-specific spatial maps {mICA ^j^(**r**)} and timecourses {mICA^j^(t))} (j = 1, 2, …, 600) were estimated using a back-reconstruction method (Calhoun et al., 2001; Erhardt et al., 2011). Then, we selected a subset of 50 components (intrinsic connectivity networks) from the 100 plenary by excluding mICAs obviously affected by physiological, motion, and imaging artifacts as characterized by noncortical activation in spatial maps and high-frequency fluctuations in timecourses (Beckmann et al., 2005; Allen et al., 2011, 2014).

The timecourses mICA(t) underwent postprocessing that included (1) detrending, (2) removing outliers, and (3) low-pass filtering with a cutoff frequency at 0.15 Hz. Finally, the postprocessed mICA(t) were normalized to have a unit variance such that the covariance matrices correspond to correlation matrices (Allen et al., 2014).

Considering the mICA as the brain functional template for group information guidance, we conducted brain functional decomposition on the group phase data δφ using the GIG-ICA method (Du and Fan, 2013), thus implementing GIG-pICA. We use the GIG-ICA method for phase data decomposition for two reasons: (1) facilitating mICA and pICA correspondence and comparison; and (2) in comparison with the direct ICA phase data decomposition (in our previous 100-subejct experiment Chen et al., 2018a) to show the convergence in phase-inferred features; for example, both pICA methods produce functional cliques in subcortical region.

The pICA timecourses were then postprocessed in ways similar to the mICA timecourse postprocessing. As a result, we obtained a set of 50 pICA components in counterpart to the 50 mICA components.

### Group mFNC and Group pFNC Matrices

According to brain structure and functional organization, we classified the 50 selected mICA components roughly into seven brain domains based on spatial activation locations, as ordered by subcortical region (SC(4)), auditory (AUD(2)), sensorimotor (SM(8)), vision (VIS(10)), cognitive control (CC(14)), default mode network (DMN(9)), and cerebellum (CB(3)).

An aggregate ICA timecourse was back-reconstructed using data from 600 subjects to generate the same number of individual subject ICA timecourses. For each subject, we calculated a temporal correlation matrix (i.e., producing a subject-specific FNC matrix). In the results, we obtained 600 single-subject {mFNC^j^} and {pFNC^j^} matrices, j = 1, 2, …, 600, for magnitude and phase data, respectively. We converted the entries in {mFNC^j^(n_1_, n_2_)} and {pFNC^j^(n_1_, n_2_)} matrices (in size of 50×50×600) to Fisher z-scores (via a Matlab routine *atanh*(*x*)). By averaging the assemblies, we obtained group-level mFNC and pFNC matrices (in size of 50×50).

### Null-Hypothesis Tests on Group mFNC and pFNC

An entry at (n_1_, n_2_) in mFNC(n_1_, n_2_) matrix represents a specific functional connection between subfunction mICA_n1_ and subfunction mICA_n2_, for n_1_, n_2_ = 1, 2, …, 50. All the entry values collected from the 600 subject-specific connections constitute an assembly of 600 samples. Through a one-sample *t*-test (on the null hypothesis that an entry at (n_1_, n_2_) in the group-level mFNC matrix assumes a zero-mean distribution across the 600 samples {mFNC^j^}), we obtained a *p*-value and an H-rest value. From all of the *t*-tests on the assembly {mFNC^j^(n_1_, n_2_), j = 1, 2,…, 600, n_1_, n_2_ = 1, 2,…,50}, we obtained a *p*-value matrix P^mFNC^ (n_1_, n_2_) in a value range [0,1] and a H-test matrix H^mFNC^(n_1_, n_2_) (binary valued {1,0}), in size of 50 × 50. In the same procedure, we obtained a *p*-value matrix P^pFNC^ and an H-test matrix H^pFNC^ from the phase data assembly {pFNC^j^}. Each entry of the *p*-value matrix was calculated from a statistic *t*-test over the 600 subject-specific FNCs with a confidence interval. The confidence intervals associated with the *p*-value matrix calculation may vary from entry to entry, which may assume different bounds delimited by positive and negative values.

The statistical hypothesis test may mistakenly produce some rejections of null hypothesis (zero mean), which we can control using the false discovery rate (FDR) through a FDR correction procedure (Benjamini and Hochberg, 1995; Benjamini and Yekutieli, 2001, 2005). This is a more powerful method for correcting FDR for multiple comparisons than the standard Bonferroni correction. It offers a strong control of the family-wise error rate (i.e., the probability that one or more null hypotheses are mistakenly rejected). The FDR correction leads to adjusted *p*-values. We made FDR corrections on P^mFNC^ and P^pFNC^ for a specified desirable FDR (default *p* = 0.05).

Based on the binary H^mFNC^(n_1_, n_2_) (H = 1 for zero-mean rejection, H = 0 for zero-mean accepted at the 5% level), we excluded the functional connections that have zero-mean distributions (entries with H = 0). We focused on the connections in mFNC(n_1_,n_2_) whose entries take on nonzero-mean distributions (determined by H = 1). We edited pFNC(n_1_, n_2_) based on the binary H^pFNC^(n_1_, n_2_).

### Numerical Characteristics of mFNC and pFNC

Based on the FDR-corrected *p*-value matrices, P^mFNC^ and P^pFNC^, we assessed the significance and robustness of the functional connections through a *p*-value thresholding as given by

(1a) $$mFNC^{<}(n_{1},n_{2})=\left\{ \begin{array}{l} \{mFNC(n_{1},n_{2}),\;{\text{P}}^{\text{mFNC}}({\text{n}}_{1},{\text{n}}_{2})<{\text{p}}_{\text{thresh}}\\ \;0,\text{ else} \end{array}\right.$$

(1b) $$pFNC^{<}(n_{1},n_{2})=\left\{ \begin{array}{l} pFNC(n_{1},n_{2}),{\text{P}}^{\text{pFNC}}({\text{n}}_{1},{\text{n}}_{2})<{\text{p}}_{\text{thresh}}\\ \;0,\text{ else} \end{array}\right.$$

where p_thresh_ denotes a specified *p*-value (p_thresh_ = 0.05 for the default statistics significance) and the superscript “<” denotes a smaller-than *p*-value thresholding. As p_thresh_ decreases, the *p*-value thresholding produces a smaller number of survival entries (≠ 0) in mFNC^<^ and pFNC^<^ matrices, representing the sparsity of higher significant functional connections; high significant connections are also strong connections.

For comparative pattern analysis of matrices mFNC^<^ and pFNC^<^, we expect the following characteristics:

1. Statistically significant connections through *p*-value thresholding in **Eq. (1a,1b)** with a span of p_thresh_ = {0.05, 10^−10^, 10^−50^, 10^−100^, 10^−150^, 10^−200^}.
2. Positive/negative connections and connectivity balance in terms of *mean*(FNC) ± *std*(FNC) (Chen et al., 2018a). The connectivity balance can be also be characterized by the entry number difference between positive count (denoted by #(+)) and negative count (denoted by #(-1)) of signs in mFNC^<^ or pFNC^<^.
3. Intra-domain (near, in a diagonal block) and inter-domain (far, in an off-diagonal submatrix) connections.
4. Sparseness and nonuniformity of significant connections. The sparsity can be numerically characterized by the small fractions #(+)/1225 and #(-)/1225, where 1225 = 50(50-1)/2 is the number of total entries in a symmetrical 50 × 50 matrix excluding the self-connections on the diagonal line. The nonuniformity is visibly inspected in mFNC^<^ and pFNC^<^ as some submatrices disappear while some other submatrices persist during the *p*-value thresholding.

## Results

### Group mFNC and pFNC

**Figure 1:**
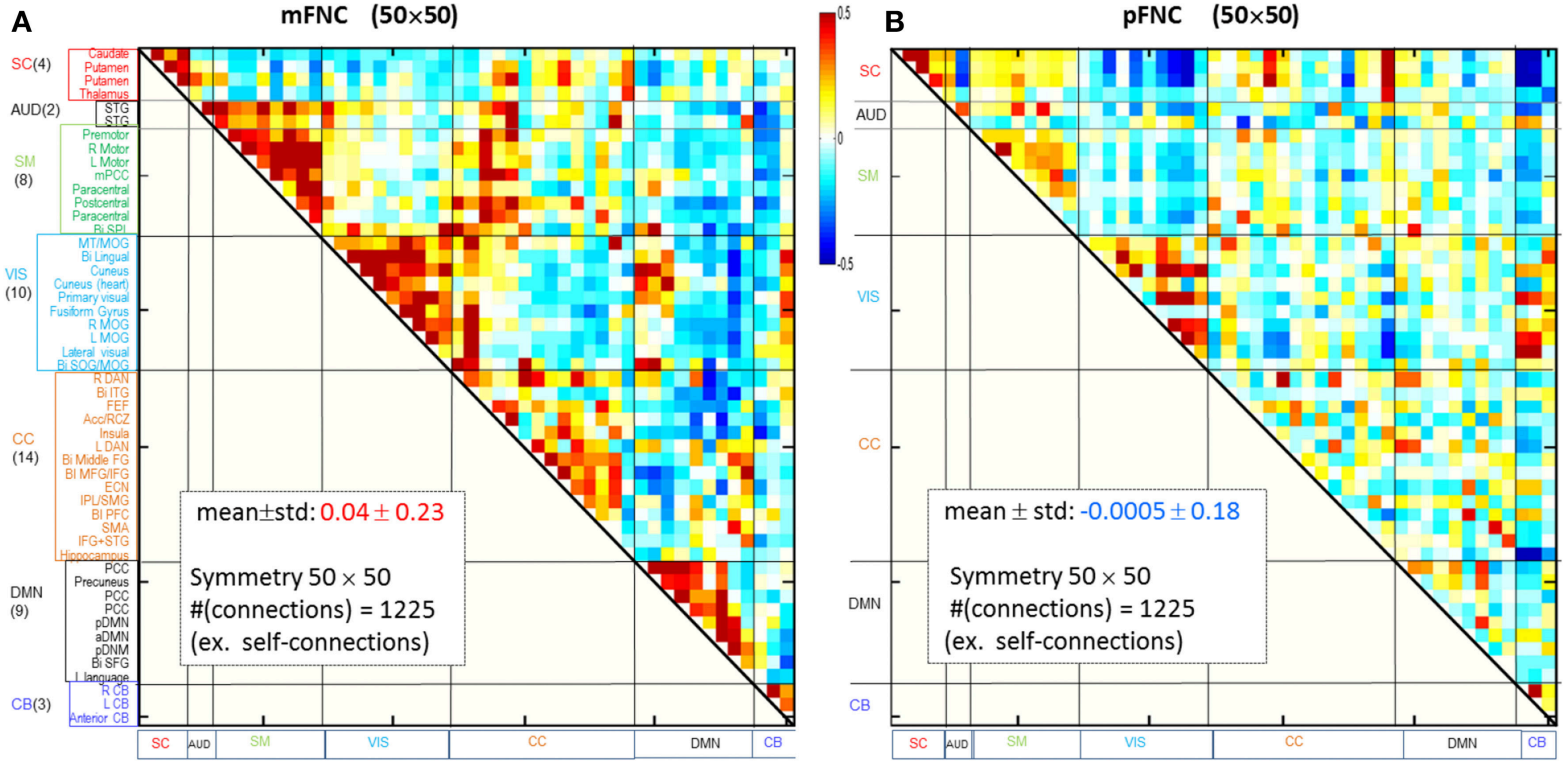
Brain resting-state subfunction arrangement and functional network connectivity. **(A)** Magnitude data depiction: a set of 50 mICA components are classified into seven (7) brain domains {SC(4), AUD(2), MOT(8), VIS(10), CC(14), DMN(9), and CB(3)} and the mFNC matrix (*mean* ± *std*: 0.04 ± 0.23); **(B)** Phase data depiction: the pICA components are classified into 7 domains and the pFNC matrix (*mean* ± *std*: −0.0005 ± 0.18).

Considering ICA components as coherent brain functional networks, we calculated the functional network connectivity matrix by the temporal correlations (Pearson correlations) among the ICA timecourses. In Figures 1A,B are shown the magnitude and phase-depicted mFNC and pFNC matrices (in size 50 × 50), as calculated by the average over the subject-specific {mFNC^j^(n_1_, n_2_)}and {pFNC^j^(n_1_, n_2_)} matrices, respectively. Note the 50 mICA components were arranged in seven domains: SC(4), AUD(2), SM(8), VIS(10), CC(14), DMN(9), and CB(3), as shown at the left vertical labels in Figure 1A. Correspondingly, the 50 pICA components were arranged with the same labels in Figure 1B.

The ICA-decomposed brain subfunctions are distributed over the brain geometrical space partitioned in seven domains. In general, the intra-domain connections are short connections, whereas the inter-domain connections are always long connections (except for rare inter-domain connections at the domain boundary). In an FNC matrix, an intra-domain short-range (near) connection constitutes the on-diagonal blocks and an inter-domain long-range (far) connection is located in the off-diagonal regions. In Figure 1, the magnitude data show strong positive near couplings in the on-diagonal blocks (Figure 1A), which differs from the phase-depicted loose connections (Figure 1B).

### One Sample *t*-tests of Group-Level mFNC and pFNC

**Figure 2:**
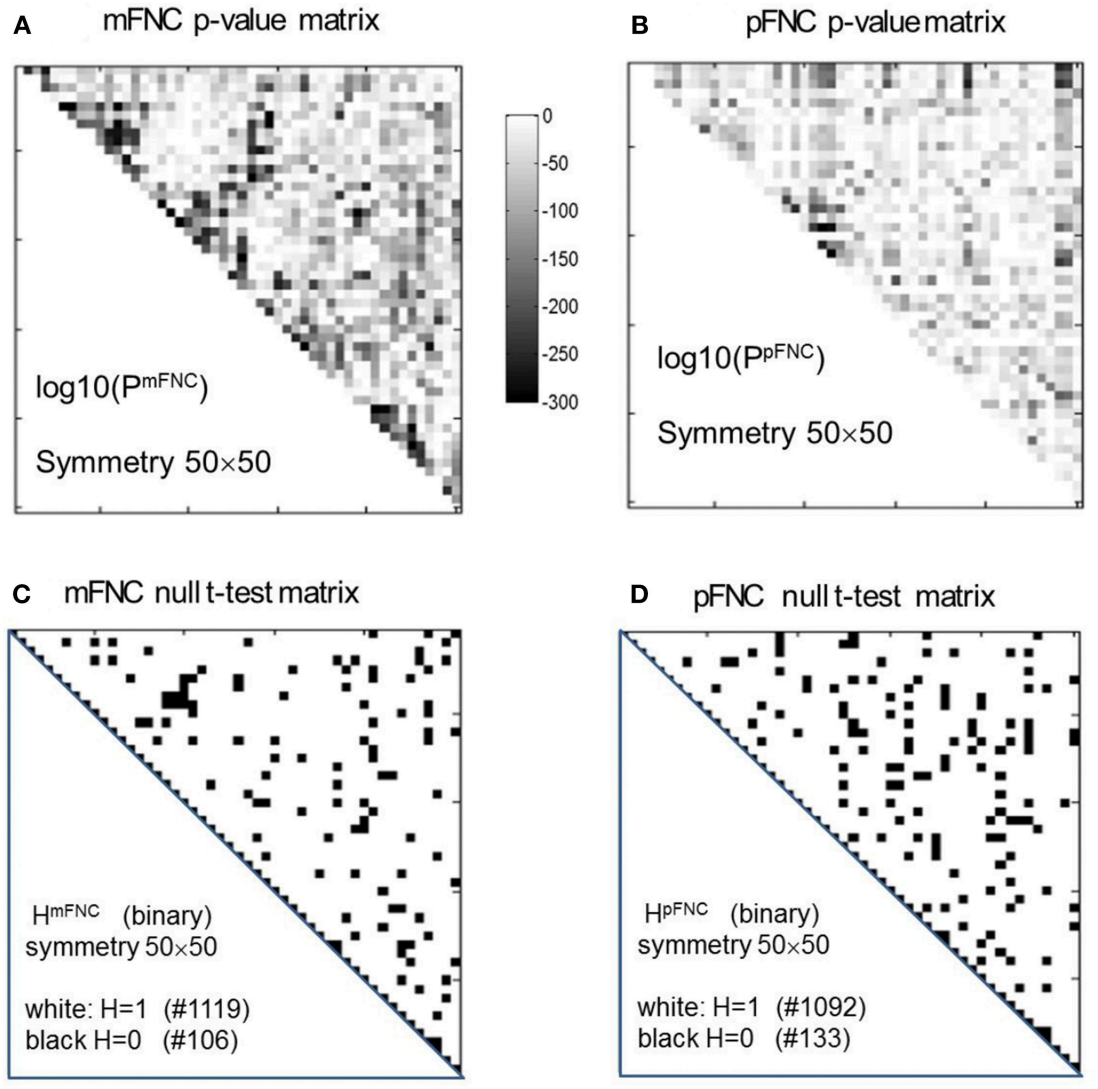
One-sample *t*-tests for the assemblies {mFNC_n_} and {pFNC_n_} (*n* = 1,2,…,600). **(A,B)** The *t*-test *p*-value matrices (displayed by a log10 magnification) using P^mFNC^(50,50) for {mFNC_n_} and P^pFNC^(50,50) for {pFNC_n_}; **(C,D)** The null-hypothesis test results (the entries with H = 1 were used for connection significance analysis).

The group-level mFNC and pFNC matrices were calculated using an average from assemblies {mFNC^j^} and {pFNC^j^}, respectively. The *t*-test on the mFNC matrix gives rise an H-test matrix H^mFNC^ and a *p*-value matrix P^mFNC^ (in size of 50 × 50), as shown in Figures 2A,C. The averaged confidence interval for P^mFNC^ is [0.02, 0.06]. Meanwhile, the pFNC *t*-test gives rise to H^pFNC^ and P^pFNC^, as shown in Figures 2B,D. The averaged confidence interval for P^pFNC^ is [−0.02, 0.02]. Note that the *p*-value matrices were displayed in a magnification by log10. The binary H-test matrices were interpreted as H = 1 for rejecting null hypothesis (nonzero mean distributions) and H = 0 for true null hypothesis (zero mean distributions).

In Figure 2C, there are 1,119 entries (H = 1 for significant connection) and 106 entries (H = 0 for noisy or random connection) in the H-test matrix H^mFNC^ (50 × 50, with a total number of 1,225 entries in the upper triangle). In Figure 2D, there are 1,092 entries (H = 1) and 133 entries (H = 0) in the H-test matrix H^pFNC^. The entries with H = 0 usually take on small values in mFNC and pFNC matrices, which we consider as noise and omit accordingly (by resetting them to zeros).

### Thresholding mFNC and pFNC

**Figure 3:**
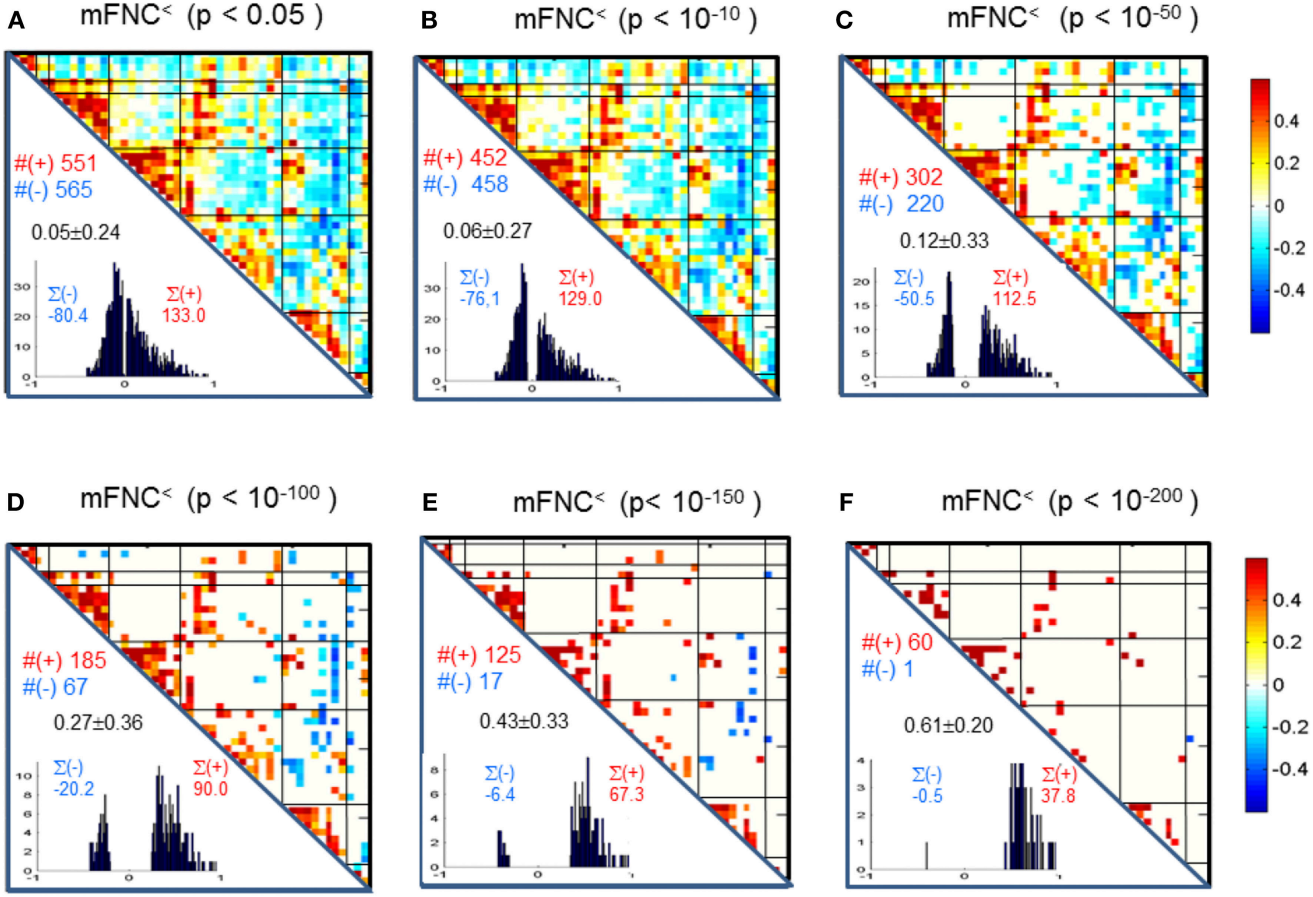
**(A–F)** Significant connections in group mFNC matrix under *p*-value thresholding with p_thresh_ = {0.05, 10^−10^, 10^−50^, 10^−100^, 10^−150^, 10^−200^}. The numbers of positive and negative connections (denoted by #(+) and #(–) respectively), and the sums of positive and negative connections (denoted by ∑(+) and ∑(–) respectively) were calculated from the survival entries in the p_thresh_-thresholded matrices (mFNC^<^).

We assessed the statistical significance of the functional connections in FNC matrices based on *p*-value thresholding in Equations (1a,b). In Figure 3, the thresholded matrices (mFNC^<^) are drawn from *p*-value thresholding with p_thresh_ = {0.05, 10^−10^, 10^−50^, 10^−100^, 10^−150^, 10^−200^} using the upper triangle portions of the symmetric matrices. For each mFNC^<^ matrix (after FDR correction), we calculated the following characteristics: *mean* ± *std*, the number of positive couplings (#(+)), the sum of positive couplings (∑(+)), the number of negative couplings (#(–)) and the sum of negative couplings (∑(–)). The connectivity balance can be characterized as *mean*(mFNC^<^). We can also quantify the functional connectivity imbalance by the quantity #(+) – #(–) in mFNC^<^ or alternatively by ∑(+) – ∑(–).

**Figure 4:**
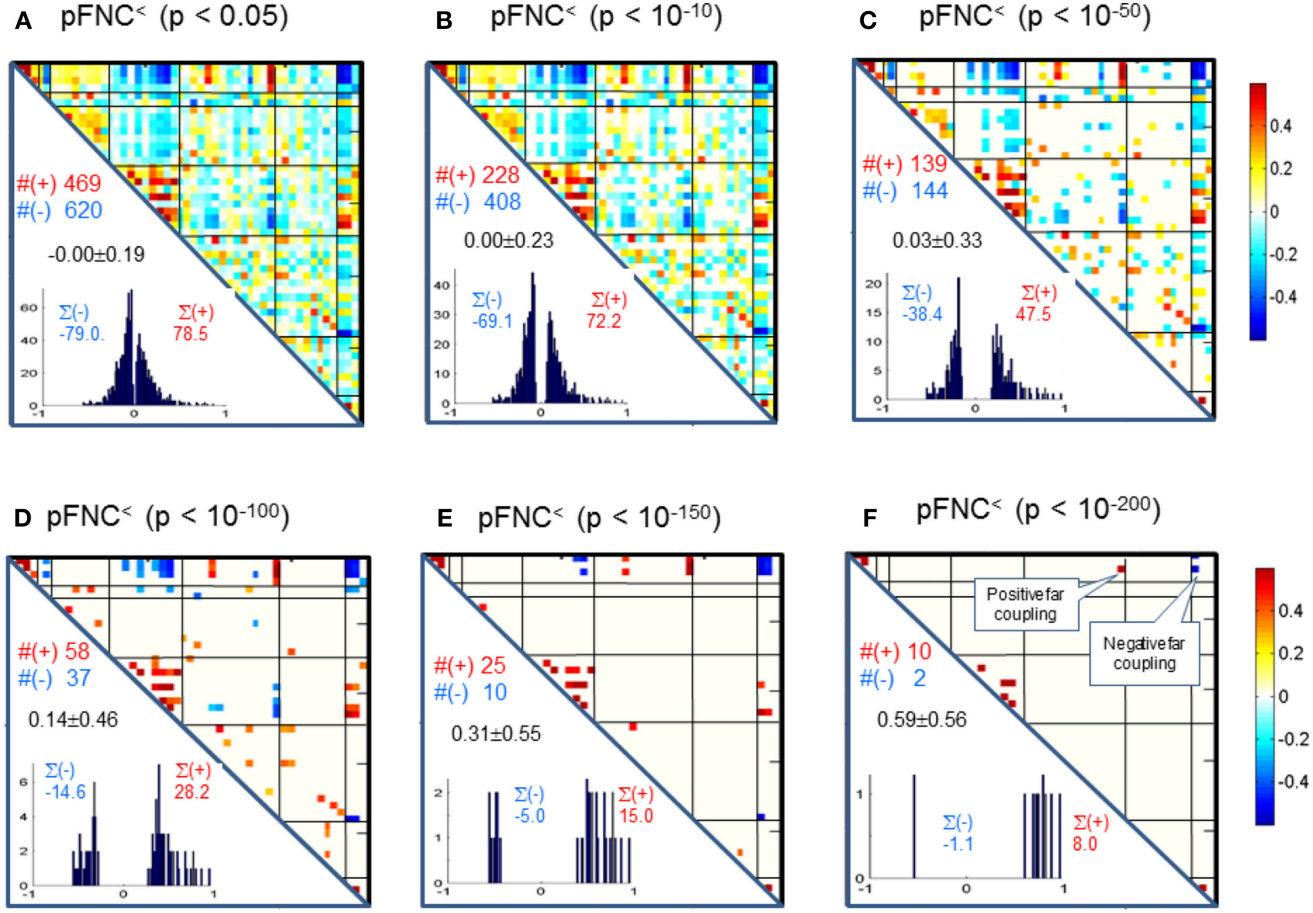
**(A–F)** Significant connections in group pFNC matrix under *p*-value thresholding with p_thresh_ = {0.05, 10^−10^, 10^−50^, 10^−100^, 10^−150^, 10^−200^}. The numbers of positive and negative connections (denoted by #(+) and #(–) respectively), and sums of positive and negative connections (denoted by ∑(+) and ∑(–) respectively) were calculated from the survival entries in the p_thresh_-thresholded matrices (pFNC^<^).

Correspondingly, in Figure 4 we show the thresholded pFNC matrices using the same *p*-value thresholdings and numerical characterizations {#(+), #(–), ∑(+), ∑(–)} as used for mFNC. In Figure 4E, the subcortical nuclei (SC) reveal significant negative couplings with VIS and CB, along with significant positive couplings with CC (*p* < 10^−150^).

**Figure 5:**
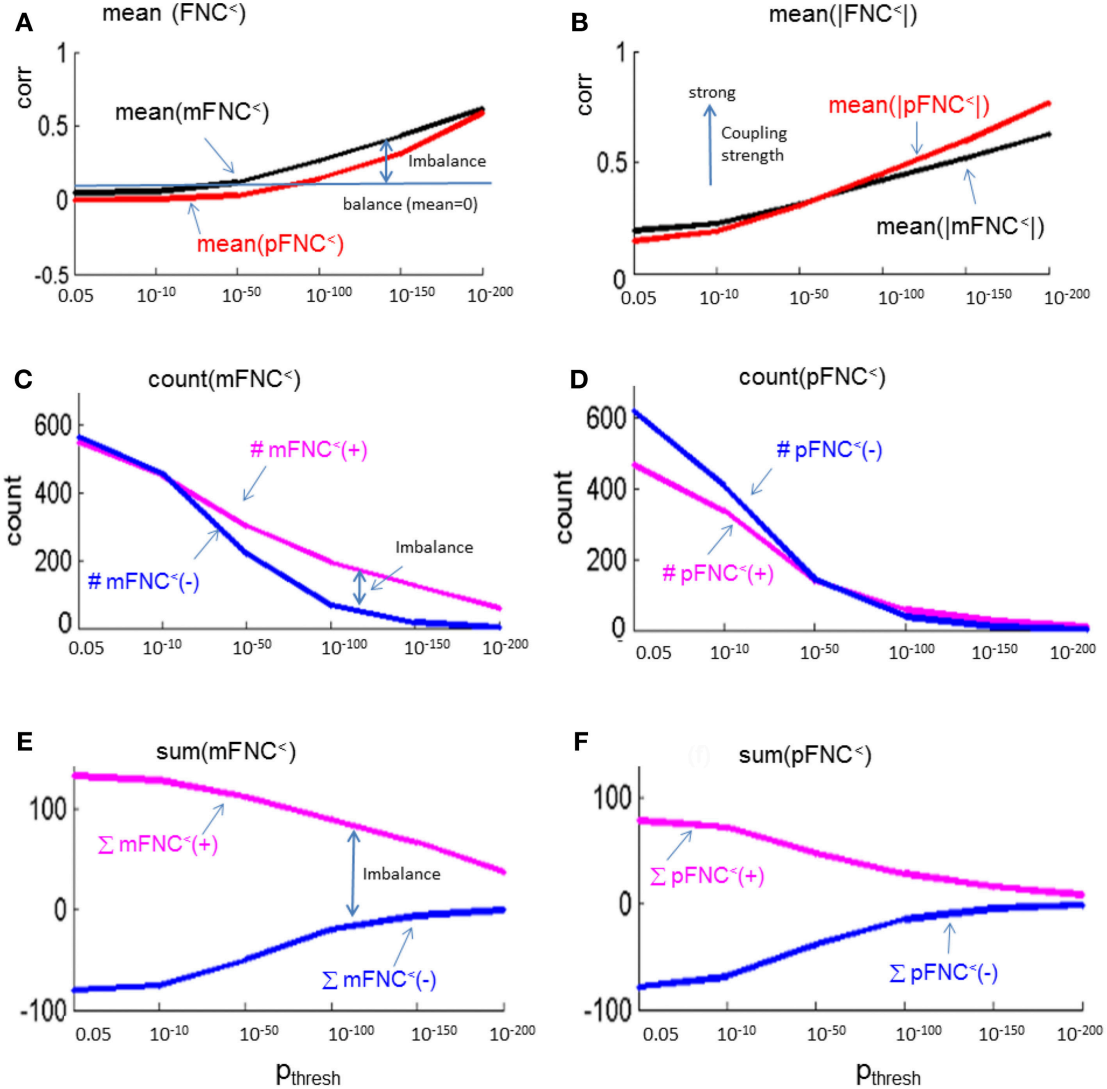
Numerical characteristics of mFNC and pFNC under *p*-value thresholding. **(A)** *mean*(mFNC^<^) and *mean*(pFNC^<^) whole-brain network balance; **(B)** *mean*(|mFNC^<^|) and *mean*(|pFNC^<^|) whole-brain network coupling strength; **(C)** Counts of positive and negative couplings in mFNC^<^; **(D)** Counts of positive and negative couplings in pFNC^<^; **(E)** Sums of positive and negative couplings in pFNC^<^; and **(F)** Sums of positive and negative couplings in pFNC^<^.

In Figure 5, we present the magnitude- and phase-depicted whole-brain connectivity behaviors for significant connections as determined by *p*-value thresholding. Specifically, we show the plots on the numerical characteristics (in terms of *mean*, counts of positive and negative connections (#(+), #(–)), and sums of positive and negative connections (∑(+), ∑(–)) of mFNC^<^ and pFNC^<^ matrices under *p*-value thresholding with p_thresh_ = {0.05, 10^−10^, 10^−50^, 10^−100^, 10^−150^, 10^−200.^}. In Figure 5A, we show the whole-brain connection balance in terms of *mean*(mFNC^<^) and *mean*(pFNC^<^), in which a large *mean* value indicates a connection imbalance (deviation from balance 0). In Figure 5B, we use the average of whole-brain connection strength in terms of *mean*(|mFNC^<^|) and *mean*(|pFNC^<^|), in which a large value indicates a strong connection. It is noted that for small *mean*(mFNC) and *mean*(pFNC) values (close to 0), we may use the *std*(mFNC) and *std*(pFNC) values to quantify connection strengths instead what was used in (Chen et al., 2018a). In Figures 5C,D, we show the positive and negative numbers (#(+) vs. #(–)) in mFNC^<^ and pFNC^<^ matrices with respect to the *p*-value thresholding, where the difference #(+) – #(–) can be used to quantify the whole-brain connection imbalance. In Figures 5E,F, we also the show the positive and negative connections in terms of ∑(+) and ∑(–) with respect to p_thresh_, where the difference ∑(+) – ∑(–) can also be used to evaluate the connection imbalance. Overall, our experimental results in Figure 5 show in comparison with fMRI magnitude data usage that the fMRI phase data reveal more connection balance (*mean*(pFNC^<^) < *mean*(mFNC^<^)) in Figure 5A, higher connection strength (*mean*(|pFNC^<^|) > *mean*(|mFNC^<^|)) in Figure 5B, and more balance in positive and negative distributions as determined by a smaller #(+) – #(–) in Figures 5C,D. and a smaller ∑(+) – ∑(–) in Figures 5E,F.

### Significant Couplings in mFNC

**Figure 6:**
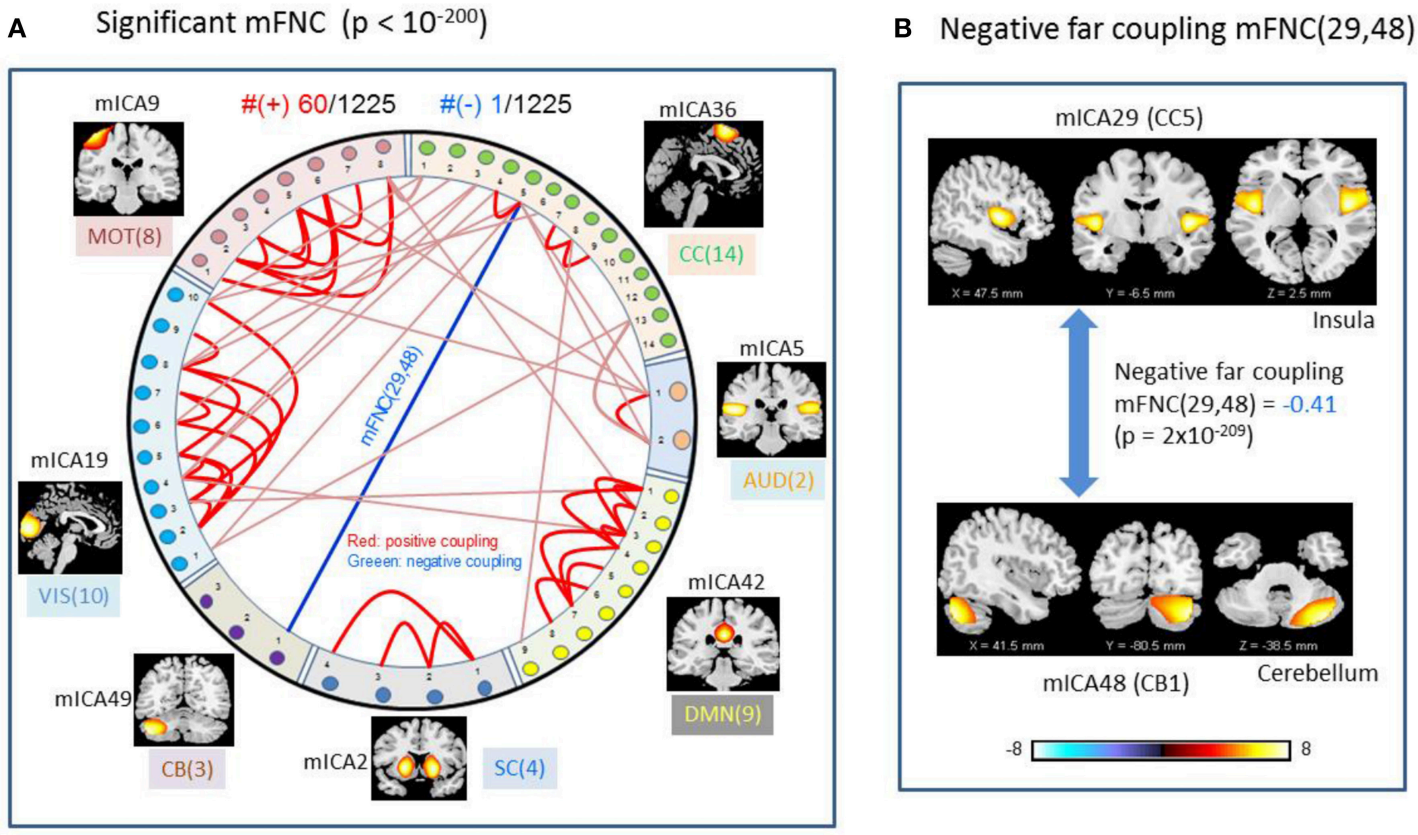
Illustrations of the most significant couplings in mFNC^<^ (*p* < 10^−200^). **(A)** There are 36 intra-domain positive couplings (near, in bright red color) and 18 inter-domain positive couplings (far, in soft red color), and 1 inter-domain negative coupling (far, in blue color). **(B)** Features of the negative interdomain coupling mFNC(29, 48).

We used *p*-value thresholding on mFNC to examine robustness and significance of the magnitude-depicted brain functional connections for whole brain space in resting state (Figure 3). Here, in Figure 6, we see significant connections survived in a very strong *p*-value thresholding (*p* < 10^−200^, Figure 3F). Specifically, we show in Figure 6A the functional connections across the seven domains (MOT(8), CC(14), AUD(2), DMN(9), SC(4), CB(3), VIS(10) in an arrangement around a circle) along with links of intra-domain (all are positive, in bright red), positive inter-domain (in dim red), and negative inter-domain (in blue). We observed the following aspects: (1) there are 60 positive connections and 1 negative connection; (2) the domains (MOT, VIS, DMN) each contain dense intra-domain connections; (3) there is no inter-domain connection between (MOT, VIS), (DMN,CC), (DMN, AUD), (DMN, SC), (DMN, CB), (SC, CB), (AUD, SC), and (AUD, CB); and (4) there is no intra-domain link in CB. In Figure 6B, we displayed the only negative connection survived in *p* < 10^−200^ during brain resting state, which shows the inter-domain connection between (CC, CB) in a connection strength 0.41 (*p* = 2 × 10^−209^). Obviously, the magnitude depicted high significant connections in Figure 6 (*p* < 10^−200^) in resting brain state are nonuniformly distributed over the brain space: dense connections in VIS, MOT, and DMN, sparse connections in CC and SC, and no connections within CB.

### Significant Couplings in pFNC

**Figure 7:**
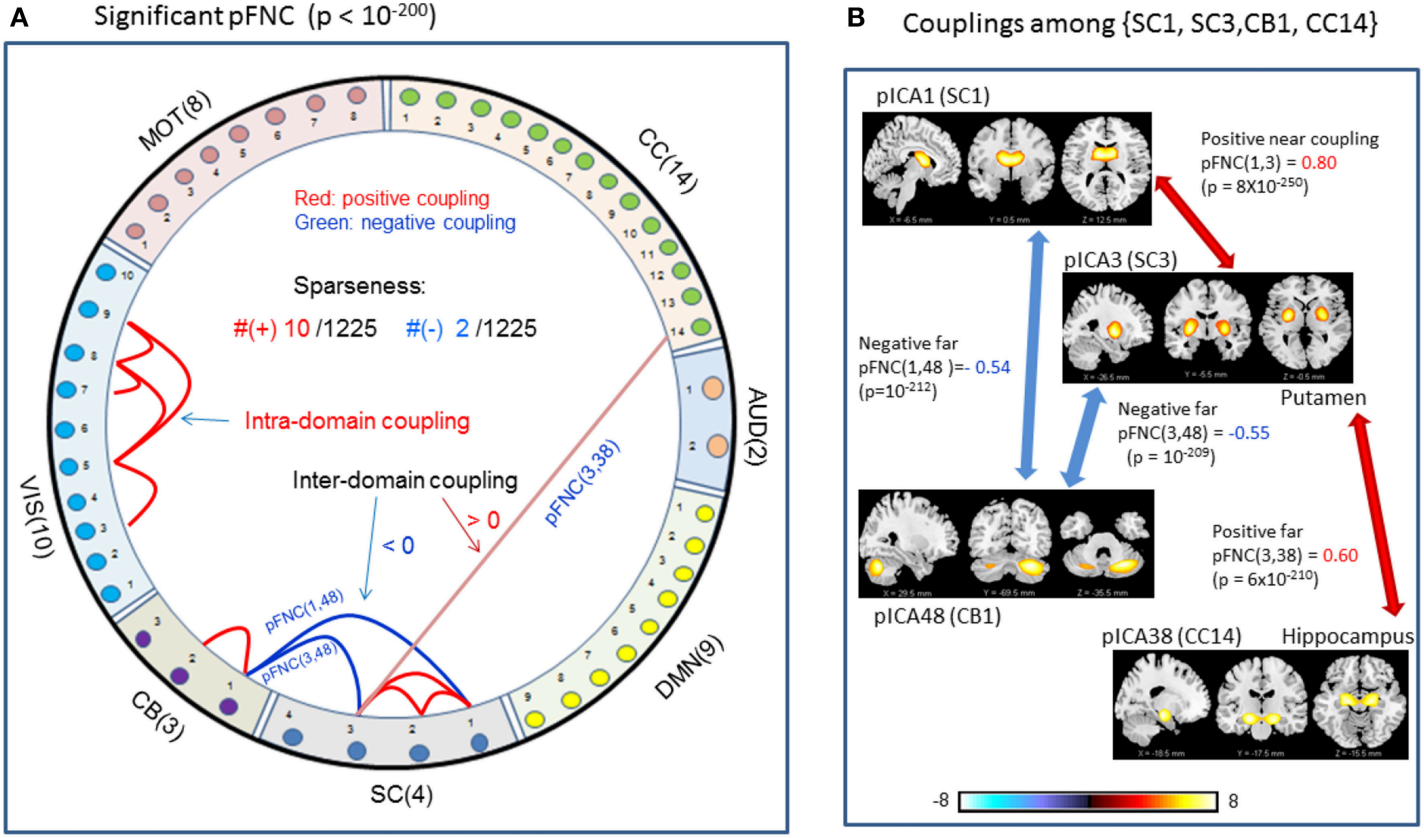
Illustrations of the most significant couplings in pFNC^<^ (*p* < 10^−200^). **(A)** There are nine (9) intra-domain positive couplings (near, in bright red color) and one (1) inter-domain positive coupling (far, in soft red color), and two (2) inter-domain negative couplings (far, in blue color). **(B)** Features of the two negative interdomain couplings pFNC(1,48) and pFNC(3, 48).

In comparison with the most significant connections in mFNC in Figure 6, we scrutinize the phase-depicted significant connections in mFNC under the same *p*-value thresholding (*p*-value < 10^−200^) in Figure 7. We observed the following aspects: (1) there are a few connections survived in *p*-value < 10^−200^ (10 positive connections and 2 negative connections); (2) there is no intra-domain connections in MOT,CC, AUD, DMN; (3) there are 2 negative far inter-domain connections between (CB, SC); (4) there is 1 positive far inter-domain connection between (CC, SC); (5) there are no inter-domain connections among {MOT, CC, AUD, DMN, VIS, CB}; and (6) only SC has inter-domain connections (1 with CC, 2 with CB). In Figure 7B, we displayed the connections among {SC1(pICA1),SC3(pICA3),CB1(pICA48)}, which assume 2 negative far inter-domain connections and 1 positive near intra-domain connection. The subcortical subfunction SC1(pFNC1) is strongly coupled with SC3(pFNC3) with pFNC(1,3) = 0.80 and a high significance (*p*-value = 8 × 10^−250^), which constitutes a functional clique in the subcortical nuclei. It seems plausible for the phase data analysis to show that the subcortical nuclei (consisting of basal ganglia and thalamus) form a functional clique that acts as a hub in couplings with other cortical subfunctions in the resting state. This observation is consistent with our previous report on the functional subcortical clique observed from a 100-subject rest fMRI experiment (Chen et al., 2018a).

## Discussion

### Inferring BOLD-Only Internal Magnetic Field Perturbation From Phase MRI

The rationale of phase fMRI for brain function study lies in the fact that we can infer the brain internal magnetic field distribution from an fMRI phase image under linear approximation (a small phase angle condition). For reference convenience, we provide the approximation theory of phase-to-field inverse mapping in the Appendix. An fMRI phase image represents a snapshot capture of the brain magnetic state (in terms of magnetic field distribution) under linear phase fMRI approximation. A voxel phase signal represents an intravoxel-average magnetic field value.

As seen in Equation (A3), phase fMRI procures a phase image φ from the MRI quadrature detection by a trigonometric operation, *arctan*(φ), which is nonlinear in a general setting. Mathematically, we have a linear approximation, *arctan*(φ) = φ for |φ| < < 1 radian (a small phase angle condition). Numerical simulation (Chen and Calhoun, 2015b) has shown that the phase fMRI nonlinearity (*arctan*(φ)) is weak for large phase angles (|φ| ~ π rad). In reality, an fMRI phase image always has phase wrapping due to the dominant phase background (|φ_0_| > π radian). In practice, an unwrapped phase image is always assumed to represent the internal magnetic fieldmap with the associated nonlinearity of large phase angles (|φ^unwrap^| > π).

For fMRI data analysis, we extract the dynamic phase perturbations (δφ) using a complex-division approach in Equation (A8), which are considered the BOLD-only phase response signals during a brain activity. Then we infer the BOLD-only magnetic field perturbation (δb) by a linear scaling mapping in Equation (A9). The small phase perturbation (typically |δφ| < 0.2 radian) ensures a good linear approximation: *arctan*(δφ) = δφ for |δφ| < < 1. Note that we cannot infer the brain magnetic fieldmap from magnitude fMRI due to irreversible nonlinearity.

### Bipolar-Valued Brain Magnetic Field Distribution

A magnetic field may assume positive and negative values. For brain fMRI study, the brain internal magnetic field is from a brain tissue magnetization in a main field B_0_. Specifically, this brain tissue magnetic susceptibility property (denoted by χ) undergoes a dipole-convolved magnetization in B_0_ to establish an inhomogeneous magnetic fieldmap. Due to the spatial derivative property of the dipole kernel, even a nonnegative susceptibility distribution (χ ≥ 0) could induce a bipolar-valued fieldmap (Chen et al., 2018b). The negative signs in a χ-induced fieldmap are maintained during the forward phase fMRI, which result in the fieldmap reconstruction by an inverse mapping from phase to fieldmap. In comparison, the negative signs are completely suppressed (inverted) in the fMRI magnitude signals due to its nonnegativeness (Chen and Calhoun, 2011). In this sense, the phase fMRI provides a direct, accurate representation of the brain magnetic state for bipolar-valued magnetic fieldmaps. Nevertheless, the magnetic fieldmap still differs from the underlying brain tissue magnetic susceptibility map using a 3D dipole convolution (Chen and Calhoun, 2013), which in principle can be completely resolved through functional quantitative susceptibility mapping (fQSM) or functional susceptibility mapping (Balla et al., 2014; Chen and Calhoun, 2015a, 2016a,c). More accurate brain functional connectivity analysis using original magnetic susceptibility source data is an important research in future.

### Positive and Negative Functional Connections

Research has shown positive and negative functional connections exist among ICA-decomposed brain networks (subfunctions) (Xu, 2015; Xu et al., 2015), either in the resting state or in task performance. Most reports on balanced connectivity (Marino et al., 2005; Fox et al., 2009; Murphy et al., 2009; Litwin-Kumar and Doiron, 2012; Liu et al., 2015) were based on fMRI magnitude data analysis in which the negative magnitude connections (anticorrelations) were reported as a result of a “de-mean” preprocessing that is prone to artifactual negative connections. Using bipolar-valued fMRI phase data, we found more negative connections that cancel the positive connection(s) to make a balanced network for the whole-brain functional connectivity without a de-mean preprocessing (Chen et al., 2018a).

Overall, the phase-depicted balanced brain functional connectivity draws from the bipolarity of phase signals (Figure 1B), while magnitude-depicted positively-biased connectivity stems from the nonnegative magnitude signals (Figure 1A). These observations are consistent with our previous report with a 100-subject experiment data analysis (Chen et al., 2018a). Since the linear inverse mapping from fMRI phase to magnetic fieldmap maintains the negative signs, the δb-depicted negative connections come from negative phase signals and anti-correlations.

### Near and Far Functional Connections

The magnitude-based brain functional connectivity study (Rosenbaum et al., 2017) has shown that nearby neurons are positively correlated, pairs at intermediate distances are negatively correlated, and distant pairs are weakly correlated. We found similar connection patterns in mFNC (Figure 1A): the on-diagonal positive blocks indicate strong near (intra-domain) connections, while off-diagonal blocks have negative and small values indicating weak far (inter-domain) connections.

In comparison, the phase-based brain function connectivity in pFNC (in Figure 1B) reveals some different patterns. Figure 1B reveals negative near connections within domains VIS, CC, and DMN in small and negative entries in the on-diagonal blocks; also seen are off-diagonal negative and positive blocks indicating strong far (inter-domain) connections. In particular, the subcortical subfunctions (SC(4)) show strong negative connections with both the visual subfunctions (VIS(10)) and the cerebellum subfunctions (CB(3)), while the VIS(10) are generally positively connected with CB(3).

### Sparseness and Nonuniformity of Brain FNC

Given a set of ICA-decomposed brain subfunctions, the whole-brain functional connectivity is numerically characterized in an FNC matrix (e.g., mFNC from magnitude data and pFNC from phase data). An entry value in the FNC matrix represents the correlation between two subfunctions in a range [−1, 1]: a large value (~1) indicates a synchrony and a negative sign an anti-correlation. The entries with small values (~0) are largely due to noise (randomness and instability). For brain function connectivity depiction, we are concerned with the strong connections (negative or positive) over the brain space (near or far connections). By omitting the entries in small values (i.e., via thresholding like Equation 1), we have a small number of survival entries in the FNC matrix showing the sparseness (counting entries in a thresholded FNC matrix) and nonuniformity of their distribution over the brain space.

In our experiment, we had a large number of subject data (*N* = 600) for statistical brain function study. Based on the FNC assemblies {mFNC^j^} and {pFNC^j^}, we conducted *t*-tests on the group-level functional connections to obtain *p*-value matrices, P^mFNC^ and P^pFNC^, and H-test matrices, H^mFNC^ and H^pFNC^, respectively. We omitted the entries in mFNC on the condition of H^mFNC^ = 0, whereby we suppress the small insignificant connection values. By using *p*-value thresholding in Equation (1a), we see significant connections as determined by the *p*-value thresholds (p_thresh_ = {0.05, 10^−10^, 10^−50^, 10^−100^, 10^−150^, 10^−200.^})shown in mFNC^<^ (see Figure 3). There are sparse (in terms of positive and negative counts) and nonuniform connections (in dense and sporadic links) in the brain space as p_thresh_ increases (as seen in Figure 6), for the most significant connections (p_thresh_ = 10^−200^). Similar sparsity and nonuniformity of phase-depicted connectivity occurs in pFNC^<^ (Figures 4, **7**). Under a specific *p*-value thresholding, the pFNC^<^ is sparser than mFNC^<^ (as indicated in #(pFNC^<^ ≠ 0) < #(mFNC^<^ ≠ 0); for p_thresh_ < 10^−50^, pFNC^<^ is more balance than mFNC^<^ in terms of |#(pFNC^<^(+) – #(pFNC^<^(–)| < |#(mFNC^<^(+) – #(mFNC^<^(–)| or |∑(pFNC^<^(+) – ∑(pFNC^<^(–)| < |∑(mFNC^<^(+) – ∑(mFNC^<^(–)| (see Figure 3 through Figure 5).

We conclude the subcortical nuclei make a functional clique (with strong intra-domain couplings) that is negatively coupled with VIS and CB subfunctions (Figure 4) while positively joined with the strongest couplings.

Significant connections are survived from *p*-value thresholding in Equations (1a,b) with a significance level specified by p_thresh_. For connection balance analysis in this particular 600-subject experiment, we suggest the use of a p_thresh_ < 10^−10^ (in Figures 3, 4). For connection sparseness analysis, we may use a very small p_thresh_ to examine a few number of high significant connections, as demonstrated in Figures 6, 7 for the extreme scenarios with p_thresh_ = 10^−200^. Comparing mFNC^<^ (Figure 3) and pFNC^<^ (Figure 4), the pFNC contains a smaller number of significant connections than mFNC and that the connections in pFNC are more balanced than those in the mFNC. In pFNC analysis, the brain resting state contains two strong negative connections between subcortical nuclei (SC) and cerebellum (CB) and the sparse intra-domain connections. Thus, basal ganglia in the SC domain have the strongest and most significant function connections with CB during brain resting state. We found one strong negative connection between CB and CC and other massive intra-domain connections (specifically #(+) = 60) from the mFNC analysis.

In neuroscience, it is well-established (Alexander et al., 1986; Amos, 2000; Stocco et al., 2010) that the central subcortex (primarily basal ganglia and thalamus) plays a “relay station” for brain functional information traffic, acting as a cohesive functional unit with strong connections to the cerebral cortex and other brain areas. Experimental data analysis (Bell and Shine, 2016) suggests the basal ganglia and thalamus are functional hubs with a core circuit supporting large-scale integration. Our 600-subject experimental results (reported herein) show strong subcortical functional cliques in the whole-brain resting state, which agree with the centralized subcortical hubs concept (Bell and Shine, 2016; Sherman, 2016; Hwang et al., 2017).

## Conclusion

Our rationale of using fMRI phase data for brain function study is based on the fact that fMRI phase imaging (unwrapped) represents the brain internal magnetic field distribution (the magnetic source for fMRI complex signal formation, the brain magnetic state at a stage prior to MRI scan and detection). We can extract the BOLD-only phase perturbation in small phase change values through calculations of the timeseries of phase images, thus ensuring a linear scale mapping to BOLD-only magnetic field perturbation (magnetic source of fMRI). Using an fMRI dataset from the cohort of 600, we compared the phase-depicted brain functional connectivity (pFNC) and the magnitude-depicted connectivity (mFNC) in terms of measures of positive and negative connections; near and far connections; sparsity and nonuniformity; and statistical significance (based one *p*-value thresholding).

Our experiments (600-subject resting-state phase fMRI) show the phase fMRI data has a smaller number of significant whole-brain connections (sparse connection) in the brain resting state than the magnitude data depiction. Perhaps, the reduced number of significant connections in phase fMRI is largely due to the positive and negative cancellation of linear phase signals. We found the basal ganglia networks (in subcortical nuclei) have strong negative connections with other brain regions in a few of the significant connections in the resting state. These findings are different from the magnitude-depicted functional connectivity in prevailing positive connections. Although we cannot prove or disprove due to a lack of *in vivo* brain function connection truth, we cannot completely confirm our findings from phase fMRI data analysis, but can justify the phase usefulness within the context of linear phase fMRI.

## Data Availability

The datasets generated for this study are available on request to the corresponding author.

## Author Contributions

All authors listed have made a substantial, direct and intellectual contribution to the work, and approved it for publication.

### Conflict of Interest Statement

The authors declare that the research was conducted in the absence of any commercial or financial relationships that could be construed as a potential conflict of interest.

## Abbreviations

BOLD: blood oxygenation level dependent
fMRI: functional magnetic resonance imaging
mICA: magnitude data independent component analysis (ICA)
pICA: phase data ICA
mFNC: magnitude-depicted function network connectivity (FNC)
pFNC: phase-depicted FNC.
