# Supplementary material for: Phase fMRI Reveals More Sparseness and Balance of Rest Brain Functional Connectivity Than Magnitude fMRI
Source: Front Neurosci. 2019 Mar 18;13:204. doi: 10.3389/fnins.2019.00204 (PMC6431653; doi:10.3389/fnins.2019.00204)
Supplement: Supplementary file 1 [file Presentation_1.pdf]

Appendix

A. Linear scale mapping between fMRI phase and internal magnetic fieldmap

A1 Complex-valued fMRI (magnitude and phase signals)

In principle, a complex-valued fMRI voxel signal is formed based on an intravoxel dephasing formula (Chen and Calhoun, 2013; Chen and Calhoun, 2012b; Haacke et al., 1999), as represented by

$$C(\mathbf{r},t)=\frac{1}{|\Omega|}\sum_{\mathbf{r}'\in\Omega(\mathbf{r})} e^{i\gamma\cdot T_E\cdot b(\mathbf{r}',t)} \quad \text{(intravoxel dephasing summation)} \qquad \text{(A1)}$$

where i=√-1 (the unit of imaginary number), b(**r**,t) is the internal spatiotemporal magnetic field distribution, T_E the echo time, γ the proton gyromagnetic ratio (a constant), Ω(**r**) a small voxel at **r** = (*x*,*y*,*z*), |Ω(**r**)| the voxel size (in a measure of a number of proton spins in a voxel space), and 1/|Ω| for signal normalization. The complex-valued voxel signal C(**r**,t) is represented by a pair of real-valued magnitude and phase signals (A(**r**,t), ϕ(**r**,t)) in a polar format or by a pair of (*real*(C), *imag*(C)) in Cartesian format, as expressed by

$$\begin{aligned} C(\mathbf{r},t) &\triangleq A(\mathbf{r},t)e^{i\phi(\mathbf{r},t)} && \text{(polar format)} \\ &\triangleq real(C(\mathbf{r},t))+i\bullet imag(C(\mathbf{r},t)) && \text{(Cartesian format)} \end{aligned} \qquad \text{(A2)}$$

The conversion between the two complex formats are expressed by

$$\begin{cases} A(\mathbf{r},t)=\sqrt{\left(real\left(C(\mathbf{r},t)\right)\right)^2+\left(imag\left(C(\mathbf{r},t)\right)\right)^2} \\ \phi(\mathbf{r},t)=\arctan\left(\dfrac{imag\left(C(\mathbf{r},t)\right)}{real\left(C(\mathbf{r},t)\right)}\right) \end{cases} . \qquad \text{(A3)}$$

In MRI technology, the quadrature detection provides a pair of (*real*, *imag*) signals, which are combined in **Eq. (A3)** to produce a pair of magnitude and phase signal (A, ϕ) in the output.

A.2 Linear phase fMRI

In order to find the relationship between the output images (A(**r**,t), ϕ(**r**,t)) and the internal fieldmap b(**r**,t), we perform Taylor expansion on the complex spin signals; that is,

$$\exp(i\gamma bT_E)=\left[1+i\gamma bT_E+\frac{(i\gamma bT_E)^2}{2!}+\frac{(i\gamma bT_E)^3}{3!}+\cdots\right]$$
$$\approx 1+i\gamma bT_E+\frac{(i\gamma bT_E)^2}{2!} \qquad (2^{nd}\text{-order approximation}) \quad . \qquad (A4)$$
$$\approx 1+i\gamma bT_E \qquad (1^{st}\text{-order approximation})$$

Through the use of approximations of $\arctan(x)\approx x$ for $|x|<<1$ and under 1st -order approximations in **Eq.(A4)**, we have

$$1^{st}\text{-order approx:} \quad \begin{cases} A(\mathbf{r},t)\approx\sqrt{1+\left(\gamma b(\mathbf{r},t)T_E\right)^2}\approx 1+\tfrac{1}{2}\left(\gamma b(\mathbf{r},t)T_E\right)^2 \\ \phi(\mathbf{r},t)\approx\arctan\left(\gamma b(\mathbf{r},t)T_E\right)\approx\gamma b(\mathbf{r},t)T_E \end{cases} \quad (\text{s.t. } |\gamma bT_E|<<1) \quad . \qquad (A5)$$

In **Eq. (A5)**, the magnitude is a nonlinear transformation in all circumstances (a quadratic mapping in the 1st-order approximation) and the phase may assume a linear transformation in the 1st-order approximation The linear approximation is subject to a small phase angle condition, $|\gamma bT_E| < 1$ radian. Under linear approximation in **Eq. (A5)**, we can infer the fieldmap from a phase image by

$$b(\mathbf{r},t)\approx\frac{\phi(\mathbf{r},t)}{\gamma T_E} \quad . \qquad (A6)$$

A.3 Brain functional modelling of fMRI

Technically, a BOLD fMRI signal is generated from the underling tissue magnetic susceptibility (denoted by χ) by a cascade of MRI transformations.(Chen and Calhoun, 2012a; Chen and Calhoun, 2015b; Chen et al., 2018b). For brain function analysis, we address BOLD-induced portions in the fMRI signals. With brain functional BOLD perturbation modelling (Chen et al., 2018b), we can describe BOLD fMRI succinctly by

$$\begin{cases} \chi(\mathbf{r},t)=\chi_0(\mathbf{r})+\delta\chi(\mathbf{r},t) & (\text{susceptibility } \chi) \\ b(\mathbf{r},t)=b_0(\mathbf{r})+\delta b(\mathbf{r},t) & (\text{fieldmap b}) \\ \phi(\mathbf{r},t)=\phi_0(\mathbf{r})+\delta\phi(\mathbf{r},t) & (\text{phase } \phi) \end{cases} \quad . \qquad (A7)$$

where the brain tissue susceptibility (χ), the χ-induced inhomogeneous fieldmap (b), the fMRI phase signals (ϕ) are all decomposed into static background and dynamic BOLD perturbations under the linear approximations of tissue magnetization and phase fMRI (Chen and Calhoun, 2012a; Chen and Calhoun, 2015b; Chen et al., 2018b). At different stages in fMRI technology, a brain BOLD activity is represented

in terms of $\delta\chi(\mathbf{r},t)$ (the origin of BOLD tissue magnetic susceptibility perturbation), $\delta\chi(\mathbf{r},t)$ (the $\delta\chi$-induced BODL fieldmap perturbation), and $\delta\phi(\mathbf{r},t)$ (the MRI-detected BOLD-only signal).

The BOLD perturbation model in **Eq. (A7)** cannot lead to an additive decomposition of the fMRI magnitude signal because of the nonlinear complex modulo operation associated with the magnitude signal formation (e.g., the nonnegative nonlinearity like $|\pm 1| = 1$) (Chen and Calhoun, 2015b). Nevertheless, the BOLD-only phase data ($\delta\chi$) is compared with the full BOLD fMRI magnitude data for brain function analysis.

A.4 BOLD-only phase data

From a timeseries of fMRI phase images $\{\phi(\mathbf{r},t_n)$, for n = 1, 2, … $N_t\}$ (a number of N_t timepoints), we can extract the dynamic relative phase change $\delta\phi$ (relative to a reference or baseline) by a complex division algorithm (Chen and Calhoun, 2016b; Chen et al., 2018b):

$$\delta\phi(\mathbf{r},t_n) = arg\left(\frac{\exp(i\phi(\mathbf{r},t_n))}{\exp(i\phi_{ref}(\mathbf{r}))}\right) \quad , \quad n = 1, 2, \cdots, N_t$$
$$with\ \phi_{ref}(\mathbf{r}) = \phi(\mathbf{r},t_1) \tag{A8}$$

where *arg* denotes an operator to find the phase angle (or argument) of a complex number (via a trigonometric function *arctan*). The reference phase image $\phi_{ref}[\mathbf{r}]$ is selected from a phase image captured at a time point (not necessarily at the 1st time point). The complex division is a phase subtraction algorithm to find the phase difference between two phase images. The static phase background $\phi_0(\mathbf{r})$ (see **Eq. (A7)**) is completely removed by the complex division in **Eq. (A8)**. An fMRI phase image is usually severely wrapped due to the dominant phase background ($|\phi_0| >> |\delta\phi|$). The BOLD perturbation is always very small (usually assuming a fraction < %5 total signal), so the relative phase difference between two phase images is free from phase wrapping phenomenon ($|\delta\phi|<\pi$). In this respect, our complex division algorithm implements phase unwrapping in the time domain (Chen and Calhoun, 2016b; Haacke et al., 2015).

From the BOLD-only phase data ($\delta\phi$), we can infer the BOLD-only fieldmap (δb) by

$$\delta b(\mathbf{r},t) = \frac{\delta\phi(\mathbf{r},t)}{\gamma T_E} \quad \text{for } |\delta\phi| \ll 1 \quad . \tag{A9}$$

Thus, δb can be inferred from $\delta\phi$ in **Eq. (A9)** with high accuracy since the $|\delta\phi| << 1$ radian is always satisfied (typically $|\delta\phi|<0.2$ radian) for a BOLD fMRI experiment. In practice, a full phase image

(unwrapped) is always used to represent the full internal fieldmap (as seen in **Eq. (A6))** despite phase imaging nonlinearity in a broad condition $|\delta\phi| \sim 1$ or $|\delta\phi| > 1$ radian.

In this appendix, we theoretically derive the linear scaling mapping between the internal magnetic field value and the fMRI phase signal based on the Taylor expansion of single procession spin signal. In practice, the fMRI phase signal formation should account for the voxel-average of the intravoxel spin signals in **Eq. (A1)** (Chen et al., 2018b). Overall, an fMRI phase image represents the voxel-averaged magnetic field distribution under linear approximation.
